# A systematic review of treatment for impulsivity and compulsivity

**DOI:** 10.3389/fpsyt.2024.1430409

**Published:** 2024-09-26

**Authors:** Beatriz Aguilar-Yamuza, Yolanda Trenados, Carlos Herruzo, María José Pino, Javier Herruzo

**Affiliations:** Department of Psychology, University of Cordoba, Cordoba, Spain

**Keywords:** systematic review, impulsivity, compulsivity, behavioral addictions, psychological treatment

## Abstract

**Introduction:**

The aim of this study was to comprehensively review existing treatments for impulsivity and compulsivity in non-substance addictions, driven by the importance of these factors in addictive disorder development and treatment efficacy.

**Methods:**

A systematic review carried out following PRISMA guidelines identified 14 articles from a total of 764 studies, highlighting the limited literature that is available on psychological treatments for non-substance addictions, in particular studies focusing on impulsivity and compulsivity. The studies were categorized by behavioral addiction type.

**Results:**

For compulsive sexual behavior and problematic pornography use, cognitive-behavioral therapy (CBT) has shown significant improvements in compulsivity. In gambling disorder, interventions like cognitive bias modification and motivational interviewing combined with CBT have been effective in reducing impulsivity. Cognitive-behavioral therapy was advocated for compulsive shopping, although results varied. For problematic internet use, dialectical behavior therapy and acceptance and commitment therapy have been effective in addressing impulsivity. Despite literature supporting CBT for most behavioral addictions, the review noted that some articles indicate that dialectical behavior therapy and acceptance and commitment therapy were also effective for problematic internet use, suggesting different underlying mechanisms for this type of addiction.

**Discussion:**

It also highlighted limitations, including the small number of studies and the lack of standardized assessment measures. Further research is needed to understand underlying mechanisms and develop tailored treatments for impulsivity and compulsivity in non-substance addictions. These findings offer new directions for research and intervention guidelines in behavioral addictions.

## Introduction

Both impulsivity and compulsivity are currently considered central constructs linked to addictive behaviors, including substance addictions and behavioral or non-substance addictions (1, 2). Impulsivity is defined as a trait that leads to unplanned, unintended, but rewarding actions that are quite inappropriate or dangerous for a given situation and often result in undesirable consequences (3, 4). Compulsivity has been defined as repetitive acts determined by a feeling that the person has to perform despite being aware that they are not in line with the overall goal (5). That is to say, compulsivity would be characterized by a lack of control over a goal-directed behavior. More specifically, compulsivity would be enhanced by the possibility of decreasing discomfort or distress, whereas impulsivity would be enhanced by desire, pleasure, enthusiasm, and gratification (6).

Both concepts have been described using different definitions in the scientific literature. For example, some phenomenological models designed to explain the origin and maintenance of addictions have postulated that addictions progress from impulsivity, characterized by positive reinforcement, to compulsivity, characterized by negative reinforcement, regardless of the possible aversive consequences of consuming a substance or of the consuming behavior itself (7, 8). However, the two types of behaviors can also share certain characteristics, such as the inability to voluntarily prevent or delay repetitive behavior, the lack of response inhibition, ineffective planning, and sensitivity to expectations of rewards (either positive or negative) (9, 10).

In contrast, Lee et al. (1) assert that compulsivity may be a broader, more multifaceted construct than impulsivity. Under this interpretation, compulsivity would also encompass an affective component that triggers an irresistible urge to behave in a certain way in order to experientially avoid the negative internal feelings that are often associated with withdrawal (11–14).

Several studies have pointed out the role played by both impulsivity and compulsivity in the origin, maintenance, and treatment of addictive behaviors (1, 9, 15, 16). Impulsivity, compulsivity and emotional dysregulation have been established as important transdiagnostic dimensions, which are relevant for comprehend both psychiatric disorders and addictive-like behaviors (1). They might be described as two ways of not being in contact with the feelings, thoughts and/or behaviors, limiting the ability to engage in goal directed or planned action (1). Impulsive actions, particularly when in distress (i.e., “negative urgency”), may be of particular relevance for the initiation of maladaptive behaviors, including addictions and addictive-like behaviors (9). Moreover, compulsivity actions, are related to entrenched behaviors in the presence of distress, stress and anxiety, potentially as these kinds of negative and assumingly dysregulated emotions, seem to increase the likelihood of overreliance on (maladaptive) habits (1). In this paper we focus on behavioral addictions. These have been defined as a particular group of addictions that do not involve the use of a psychoactive substance (17, 18). Like substance addictions, however, behavioral addictions are clinical entities involving a series of impulsive, repetitive behaviors that would appear to be characterized by: loss of control over the behavior in question, a strong compulsive desire to engage in the behavior, and emotional discomfort or distress that leads to persistence in the behavior despite the aversive consequences it may have (19).

The Diagnostic and Statistical Manual of Mental Disorders (DSM-5-TR) (20) includes a preliminary mention of certain behavioral addictions, such as exercise addiction, shopping addiction and sex addiction, but the only behavioral addiction it recognizes as a clear diagnostic entity is gambling disorder (20). On the other hand, the International Classification of Diseases (ICD-11) not only includes gambling disorder (under the heading *Disorders due to addictive behaviors*) but also recognizes other behavioral addictions diagnosable as *Other Specific Impulse Control Disorders* (21). Although there are many other excessive and problematic behaviors which can be understood as potentially addictive behaviors (compulsive shopping, problematic use of the Internet or social networks, etc.), these are not included in the DSM-5 or ICD-11. They are, however, mentioned in the scientific literature (17) or included in guidelines (22). Indeed, pre-existing levels of impulsivity and compulsivity have been identified as indicators of an increased risk of developing addictive problems (16, 23, 24). People who show a higher level of both impulsivity and compulsivity, for example, also present higher levels of problematic Internet use (23, 25), gambling disorder (26, 27), compulsive buying disorder (28, 29) and exercise addiction (30, 31).

It should also be emphasized that, depending on the behavioral addiction in question, differences can be appreciated between levels of impulsivity and compulsivity. Gambling disorder dependence, for example, has been consistently linked to high levels of impulsivity and compulsivity when performing different types of neurocognitive tasks (1, 32). More specifically, people with pathological gambling habits tend to show deficits in response inhibition, attentional set shifting, and contingency-related cognitive inflexibility tasks (1). Sensation seeking has also been found to be a good predictor of gambling (33). With regard to other behavioral addictions, however, little literature can be found that relates them to phenomena like impulsivity or compulsivity. Since they have, to date, been studied less frequently, no measures have yet been obtained which address compulsivity in those disorders (1). Even less information is currently available on the most appropriate treatment with which to address phenomena such as impulsivity and compulsivity in behavioral addictions (34).

Current psychosocial treatments for addictive disorders, such as cognitive-behavioral therapy, 12-step programs, or motivation-focused programs, have been found to have certain limitations in addressing impulse control (35, 36). Likewise, although cognitive remediation represents a promising complementary approach for improving impulse control in addictive disorders (37, 38), its efficacy remains unproven. A considerable amount of research has highlighted third generation treatments or contextual therapies, including a variety of psychotherapies for addressing and reducing impulsivity among people with addictive substance use disorders (39–42). However, although the number of studies on treatments that reduce the level of impulsivity and compulsivity in substance use disorders has increased in recent years, scientific works focused on behavioral addictions are scarce (34, 43). Finally, the systematic reviews found to the date (34, 44, 45) only address treatment for a specific behavioral addiction, without providing a generalized summary of the current situation with respect to other non-substance addictions. According to the results of these reviews, the treatments of choice seem to be: cognitive-behavioral treatment and motivational interviewing combined with cognitive-behavioral therapy; which are in line with the previous data. For this reason, this study proposes a review that gathers all the information about the treatments in force for all behavioral addictions to date.

In view of the facts that 1) both impulsivity and compulsivity are important factors in the onset, maintenance and treatment of addictive disorders (27, 46); 2) psychological intervention produces significant improvements in a person’s impulsivity, compulsivity and quality of life (47); and 3) the literature available on the psychological approach to impulsivity and compulsivity in behavioral addictions is so scarce, the aim of the present study was therefore to conduct a comprehensive literature search and review of the different existing treatments for both constructs in non-substance addictions.

## Method

To find scientific publications addressing the psychological treatment of impulsivity and/or compulsivity in behavioral addictions, a systematic review methodology following the PRISMA model (46) was used. Four online searches were conducted on March 8, 2024, in each of the following databases: PubMed, Cochrane Library, Web of Science, and Scopus. The search strategy used the terms and Boolean operators shown in **Table 1**. No filters were applied and the search was performed by title, abstract and keywords.

**Table 1:** Search strategy.

Search	Search criteria used in each of the databases
1	(“Behavioral Addiction”[Mesh]) AND “Impulsivity”[Mesh] AND “Compulsivity”[Mesh] AND “Treatment”[Mesh]).
2	(“Behavioral Addiction”[Mesh]) AND “Impulsivity”[Mesh] AND “Treatment”[Mesh]).
3	(“Behavioral Addiction”[Mesh]) AND “Compulsivity”[Mesh] AND “Treatment”[Mesh]).

### Inclusion and exclusion criteria

Inclusion criteria were: (1) scientific articles; (2) quantitative empirical results/data; and (3) psychological treatment of impulsivity and/or compulsivity in behavioral addictions. Exclusion criteria were: (1) single case studies; (2) qualitative results; (3) descriptive results; (4) pharmacological treatment; and (5) substance addictions. It should be clarified that, although all publications date from 2020 to 2023, the criterion used was not that the articles should be recent.

### Selection of articles

**Figure 1:**
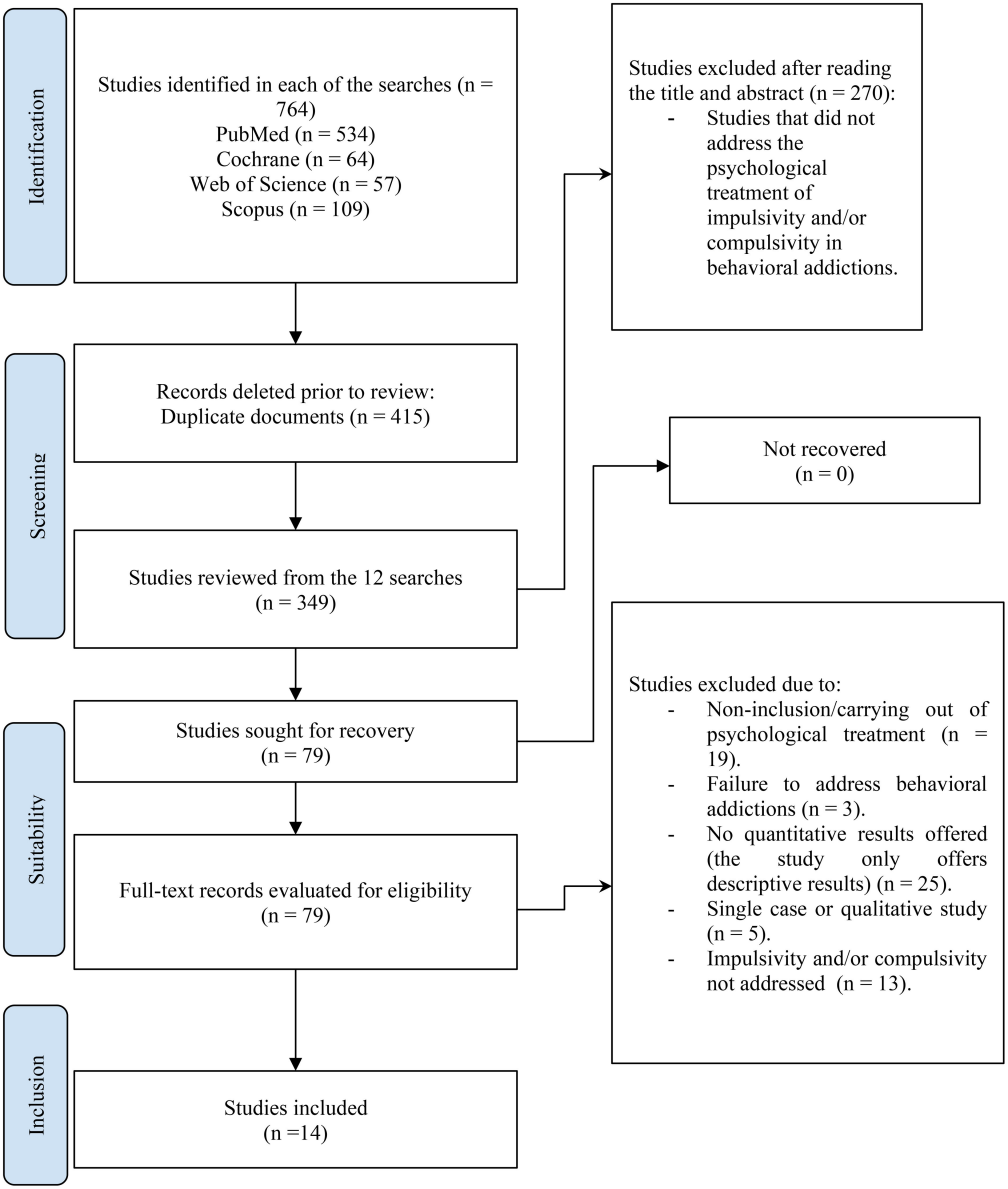
PRISMA flow chart.

Articles were selected in accordance with the PRISMA guidelines (48) (see **Figure 1**). Out of a total of 349 articles, 270 were rejected after review of the abstract or title. A total of 79 articles were selected for detailed full-text review. The selection procedure was carried out independently by two researchers. When there was a disagreement, a third party was involved to resolve it. The final sample comprised 14 articles that met the inclusion criteria and all of the exclusion criteria referred to in the previous section.

### Extraction of results

The characteristics of the 14 selected studies are shown in **Table 2**.

**Table 2:** Descriptive characteristics of the articles selected, in alphabetical order (n = 14).

Reference	Location	Sample (proportion of women)	Type of problem	Type of treatment	Behavior/trait addressed	Results	Quality score
Antons et al. (44)	Germany	N = 24	Compulsive sexual behavior (CSBD) and problematic pornography use (PPU).	Psychotherapy based on cognitive-behavioral treatment	Compulsiveness	General improvement of symptomatology associated with PPU and CSBD and significant improvements in compulsivity.	–
Boumparis et al. (34)	Switzerland	N = 29	1) Gambling disorder (pathological gambling). 2) Problematic use of pornography.	1) Intervention aimed at modifying cognitive bias. 2) Motivational interviewing combined with cognitive-behavioral therapy.	Impulsivity	A reduction in impulsivity levels, measured using the Barratt Impulsiveness Scale (BIS), was observed in both problems.	–
Holas et al. (50)	South and North Dakota	N = 13 (0% female)	Compulsive sexual behavior	Relapse prevention based on mindfulness.	Compulsiveness	Compulsivity was measured with the Obsessive-Compulsive Inventory-Revised (OCI-R). *r* = .43; mean effect (Cohen, 1988).	Regular
Garcia-Caballero et al. (47)	Spain	N = 18 (0% female)	Gambling disorder (pathological gambling).	Motivational interviewing and cognitive behavioral therapy.	Impulsivity	Impulsivity was measured with the Impulsive Behavior Scale (UPPS-P). - Negative urgency: p = .001 - Positive urgency: p = .001 - Lack of premeditation: p = .029 - Lack of perseverance p = .048	Regular
He et al. (59)	China	N = 48 (81.25% female)	Problematic use of online gambling.	Bias modification treatment of the stimulus-response compatibility approach.	Impulsivity	Impulsivity was measured with the Barratt Impulsiveness Scale (BIS). None of the results showed significance.	Good
Kim et al. (51)	Canada and the United States	N = 185 (43.2% women)	Gambling disorder (pathological gambling)	Simultaneous addiction substitution and/or recovery	Impulsivity	Impulsivity was measured with the Impulsive Behavior Scale (UPPS-P). - Negative urgency: p = .038 - Lack of premeditation: p = .040	Good
Mestre-Bach et al. (52)	Spain	N = 77 (100% women)	Compulsive shopping.	Cognitive-behavioral treatment.	Compulsivity	Compulsivity was measured with the SCL-90-R. - Obsessive/compulsive dimension: No significant improvements were found (p= .267), obtaining a small effect size (|d| = .28).	Good
Müller et al. (45)	Germany	N = 13	Compulsive shopping.	Cognitive-behavioral treatment.	Impulsivity and compulsivity	A reduction in the levels of impulsivity and compulsivity, measured with the ICD-SCID and Richmond-CBS questionnaires, respectively, was observed in both patients.	–
Na et al. (53)	South Korea	N = 20 (75% women)	Excessive use of internet and/or computer games	Acceptance and commitment therapy.	Impulsivity	A significant decrease in impulsivity was observed. (p = .018).	Good
Romo et al. (54)	France	N = 1423 (53% women)	Problematic use of social networks	Cognitive-behavioral therapy	Impulsivity	Impulsivity was measured using the Impulsive Behavior Scale (UPPS-P) and the results showed no significance.	Good
Shahrajabian et al. (55)	Iran	N = 36 (69.45% female)	Problematic use of the Internet.	Emotional working memory training (eWMT)	Impulsivity and compulsivity	A positive effect was seen on the ability to inhibit impulsivity. - Time: p <.001; n2 = .78 - Group: p <.001; n2 = .58 - Time x group: p <.001; n2 = .71.	Good
Siste et al. (56)	Indonesia	N = 40 (57.5% women)	Problematic use of the Internet.	Dialectical behavioral therapy	Impulsivity	A very significant decrease in impulsivity was observed. (p <.001).	Good
Zhang et al. (57)	China	N = 18 (66.67% female)	Problematic use of the Internet.	Solution-focused group counseling intervention.	Compulsivity	A significant decrease in compulsivity was observed. (p <.01).	Regular
Zhao & Pan (58)	China	N = 100 (32% women)	Problematic Internet use (referred to in the article as Internet addiction).	Psychosocial intervention.	Compulsivity	Compulsivity decreased significantly, as the scores obtained in the CIAS-R by the experimental group were significantly lower than those obtained by the control group. (p <.05).	Good

### Quality assessment

The quality of the articles was assessed using the Quality Assessment Tool for Cohort and Cross-Sectional Observational Studies (49). 78.57% (n = 11) of the articles were assessed by both authors. Once the researchers had come to an agreement regarding discrepancies (97.2%), the quality assessment was deemed complete.

The articles were classified as being of “good”, “fair” or “poor” quality according to an overall judgment based on the 14 criteria proposed by the Quality Assessment Tool for Cohort and Cross-Sectional Observational Studies. These criteria, which are shown in **Appendix: Annex 1**, were not applicable to 3 of the 14 articles (1, 2 and 8) because they were systematic reviews.

## Results

The different sections below, established according to the type of behavioral addiction being addressed, describe the different interventions carried out in each case. That is to say, to ensure that the results are clearly presented, the different studies found are grouped together according to the clinical problems they address.

### Compulsive sexual behavior and problematic pornography use

Three articles were found that addressed compulsive sexual behavior. The first of these was a systematic review by Antons et al. (44). This included a total of 24 articles addressing the treatment of compulsive sexual behavior and problematic pornography use. In its results, the study reveals clinically significant improvements in compulsivity mainly when psychotherapy based on cognitive-behavioral treatment was applied. The second study, by Holas et al. (50), only addresses compulsive sexual behavior in relation to a mindfulness-based relapse prevention program. According to the results obtained, the program in question would also produce improvements in compulsivity with a medium effect size (*r* = .43). Finally, the systematic review conducted by Boumparis et al. (34) focused on the problematic use of pornography. In their results, the authors state that motivational interviewing combined with cognitive-behavioral therapy produced a reduction in impulsivity levels, which were measured pre- and post-intervention using the Barratt Impulsiveness Scale (BIS).

### Gambling disorder

Three articles addressing gambling disorder were included. The first, by Boumparis et al. (34), found that an intervention aimed at modifying cognitive bias produced improvements in impulsivity levels. The second revealed that motivational interviewing conducted together with cognitive-behavioral therapy produced improvements in impulsivity (47). Impulsivity was measured using the Impulsive Behavior Scale (UPPS-P) and significant differences were found in the following dimensions of that scale: 1) negative urgency (*p* = .001); 2) positive urgency (*p* = .001); 3) lack of premeditation (*p* = .029); and 4) lack of perseverance (*p* = .048). The third article also reported improvements in impulsivity when working with addiction substitution and/or concurrent recovery (51). Here, improvements were observed in the following dimensions: 1) negative urgency (*p* = .038); and 2) lack of premeditation (*p* = .04).

### Compulsive shopping

Two articles addressing compulsive shopping were included. Both of them advocate cognitive-behavioral therapy as the treatment of choice. The first, which measured compulsivity based on the Obsession/Compulsion dimension of the SCL-90-R, did not, however, find significant improvements in compulsive behavior (*p* = .267) (52) and the effect size obtained was small (*d* = .28). For its part, the study by Müller et al. (45) focused on both compulsive and impulsive behavior. This article was actually a systematic review. Its results indicated that cognitive-behavioral therapy was the treatment of choice for addressing compulsive shopping, producing a reduction in levels of impulsivity (measured with the ICD-SCID) and compulsivity (measured with the Richmond-CBS).

### Problematic use of internet/social networks

Six articles addressing problematic use of the Internet or social networks were included. The first, authored by Na et al. (53) reported significant improvements in impulsivity through intervention with acceptance and commitment therapy (*p* = .018). The second, focusing on the cognitive-behavioral treatment of impulsivity, found no significant improvements (54). The third, by Shahrajabian et al. (55) analyzed the effect of emotional working memory training (eWMT) treatment on impulsivity and compulsivity. However, significant improvements were only found in impulsivity, a large effect size being observed when both groups were compared with each other (n^2^ = .58) and when the same group was compared over time (n^2^ = .78). The evolution of both groups over time was analyzed and the effect size was again large (n^2^ = .71). The fourth study addressed problematic Internet use through dialectical behavioral therapy (56). In their results, the authors observed a highly significant decrease in impulsivity (*p* <.001). The fifth article, by Zhang et al. (57), described a solution-focused group counseling intervention resulting in a significant decrease in symptomatology associated with compulsive behavior (*p* <.01). The last study reported a significant decrease in compulsivity (*p* <.05) after having carried out a psychosocial intervention consisting of a cognitive-behavioral group therapy lasting three months During that time, group cohesion and mutual support were promoted as a central pillar for helping members achieve an adequate use of the Internet (58).

### Problematic use of online gambling

Only one of the studies included focused on problematic online gambling. In it, the disorder was addressed through the bias modification treatment proposed in the stimulus-response compatibility approach. The results obtained showed no significant differences between the experimental group and the control group after treatment (59).

## Discussion

The present systematic review involved a comprehensive literature search and review of the different existing treatments for impulsivity and compulsivity in non-substance addictions. After performing the search, a total of 764 studies were found, of which only 14 met the previously established inclusion and exclusion criteria. Regardless of these criteria, attention should be drawn to the small number of studies that have been published on psychological treatments for non-substance addictions that decrease levels of impulsivity and compulsivity.

The articles identified covered different psychological treatments for impulsivity and/or compulsivity in the following behavioral addictions: compulsive sexual behavior, problematic use of pornography, gambling disorder (pathological gambling), compulsive shopping, excessive/problematic use of the Internet and/or computer gaming, and problematic use of social networks.

Three of the fourteen studies found were systematic reviews that referenced studies into treatment for compulsive sexual behavior and problematic pornography use. They also assessed the effects of treatment on symptom severity and behavioral activation, systematically evaluated the broader literature on Internet-based interventions targeting behavioral addictions, and provided a systematic update on studies into treatment for compulsive sexual behavior. Overall, the results of these three studies showed reductions in levels of impulsivity and compulsivity through the respective use of cognitive-behavioral therapy, cognitive bias modification and motivational interviewing combined with cognitive-behavioral therapy, and, again, cognitive-behavioral therapy.

The systematic review therefore offers promising results regarding the treatment of impulsivity and/or compulsivity in the following problems: compulsive sexual behavior, problematic use of pornography, gambling disorder, compulsive shopping, problematic use of the Internet and/or social networks, and problematic use of online gambling. According to the data sowed in the articles included in this systematic review, the treatment of choice for compulsive sexual behavior and problematic pornography use would be cognitive-behavioral treatment. For impulsivity in gambling disorder, the treatment with the greatest empirical support would be the use of motivational interviewing together with cognitive-behavioral therapy. For the treatment of compulsive shopping, the results were contradictory: the study by Mestre-Bach et al. (52) found no significant improvements after a cognitive-behavioral intervention, while the review by Müller et al. (45) proposes cognitive-behavioral therapy as the treatment of choice for this problem. In relation to problematic Internet use, the two therapies postulated as the most suitable for treating impulsivity are dialectical behavior therapy and acceptance and commitment therapy, the former being slightly more effective. Emotional working memory training (eWMT), solution-focused group counseling intervention and psychosocial intervention are also postulated in the literature as probably effective. Finally, for problematic online gambling only one study was found that proposed stimulus-response bias modification treatment, a therapy which apparently does not produce significant improvements in either impulsivity or compulsivity.

Additionally, the following findings could be extracted from the literature reviewed: the most analyzed and effective treatment was cognitive-behavioral therapy for all behavioral addictions, being also the treatment that showed controversial results in problematic internet use; the bias modification treatment of the stimulus-response compatibility approach for the problematic use of social networks did not show evidence of effectiveness; the most studied problem was problematic internet use, being addressed by a total of 6 of the 14 articles included. Based on these findings, it would be interesting to investigate other types of treatments that have not yet been sufficiently explored in the field of behavioral addictions such as: brief therapy in primary care and/or family therapy, since most of the patients with problematic internet use, video game addiction or other types of behavioral addictions are young people.

The results detailed in the previous paragraph are consistent with the existing literature on treatments for behavioral addictions (60–62), since they indicate that motivational interviewing together with cognitive-behavioral therapy would be the treatment of choice for different non-substance addictions (47, 51). Surprisingly, however, although cognitive-behavioral therapy appears in the literature as the most effective method for addressing problematic internet use (63–65), in our review it offers no significant improvements in impulsivity levels (54). In fact, our results postulate third-generation therapies (dialectical behavioral therapy and acceptance and commitment therapy) as preferred options for addressing this trait (53, 56).

It should be noted that this review is not without its limitations. First, the number of studies included was not very high and this may limit the conclusions that can be drawn from the literature. Also, the included studies featured no common assessment measure for both impulsivity and compulsivity, while sample heterogeneity with respect to gender was observed in only half of the studies analyzed. Also, it is important to mention that, although qualitative studies have been excluded in this article, it would also be interesting to examine the existing literature form a nomothetic perspective and consider individual biographies, current circumstances and life projects for personalized treatment. Finally, most of the studies included focused on only one of the two constructs, the exceptions being the two papers mentioned in **Table 2**, which assess both impulsivity and compulsivity.

This systematic review was conducted in order to determine the treatments for impulsivity and/or compulsivity in non-substance addictions. The results obtained identify cognitive-behavioral therapy as the treatment of choice for most behavioral addictions (compulsive shopping, compulsive sexual behavior, problematic pornography use and gambling disorder). This is in line with what is found in the scientific literature (62). With regard to problematic internet use, however, the therapies that are postulated as effective for reducing impulsivity do not concur with the findings reported in the scientific literature: in this review dialectical behavioral therapy and acceptance and commitment therapy obtained the best results (53, 56). This may suggest that the underlying, explanatory mechanisms of problematic Internet use and the impulsivity associated with it are not the same as those of other, conventional addictions. In fact, the results seem to indicate that this type of problem may be more responsive to transdiagnostic processes such as experiential avoidance (which are mainly addressed by third-generation therapies) as opposed to other types of typical addiction characteristics such as craving. Due to the small number of studies included in this review (*N* = 14), however, further research on these types of problems is needed in order to identify the mechanisms underlying impulsivity and compulsivity and to determine the most effective treatments for these traits.

These results open new horizons for research into treatments for impulsivity and/or compulsivity in non-substance addictions and can also serve as a guide when establishing and proposing future intervention guidelines for use with behavioral addictions.

The authors report no declarations of interest.

## Data Availability

The original contributions presented in the study are included in the article/supplementary material. Further inquiries can be directed to the corresponding author.

## Appendix: Annex I

Access to the checklist criteria of the “Quality Assessment Tool for Cohort and Cross-Sectional Observational Studies” (National Heart, Lung and Blood Institute, 2014). Study Quality Assessment Tools | NHLBI, NIH.

1. Research objective: Was the research question or objective in this paper clearly stated?
2. Study population: Was the study population clearly specified and defined?
3. Participation rate ≥50%: Was the participation rate of eligible persons at least 50%?
4. Recruitment: Were all the subjects selected or recruited from the same or similar populations (including the same time period)? Were inclusion and exclusion criteria for being in the study prespecified and applied uniformly to all participants?
5. Sample size: Was a sample size justification, power description, or variance and effect estimates provided?
6. Exposure before outcome: For the analyses in this paper, were the exposure(s) of interest measured prior to the outcome(s) being measured?
7. Timeframe: Was the timeframe sufficient so that one could reasonably expect to see an association between exposure and outcome if it existed?
8. Levels of exposures: For exposures that can vary in amount or level, did the study examine different levels of the exposure as related to the outcome (e.g., categories of exposure, or exposure measured as continuous variable)?
9. Exposure measurement: Were the exposure measures (independent variables) clearly defined, valid, reliable, and implemented consistently across all study participants?
10. Exposure assessment in time: Was the exposure(s) assessed more than once over time?
11. Outcome measurement: Were the outcome measures (dependent variables) clearly defined, valid, reliable, and implemented consistently across all study participants?
12. Blindness: Were the outcome assessors blinded to the exposure status of participants?
13. Loss to follow-up ≤20%: Was loss to follow-up after baseline 20% or less?
14. Confounding: Were key potential confounding variables measured and adjusted statistically for their impact on the relationship between exposure(s) and outcome(s)?
